# Supplementary figures and images for: Decoding the Different Aroma-Active Compounds in Soy Sauce for Cold Dishes via a Multiple Sensory Evaluation and Instrumental Analysis
Source: Foods. 2023 Oct 8;12(19):3693. doi: 10.3390/foods12193693 (PMC10572970; doi:10.3390/foods12193693)

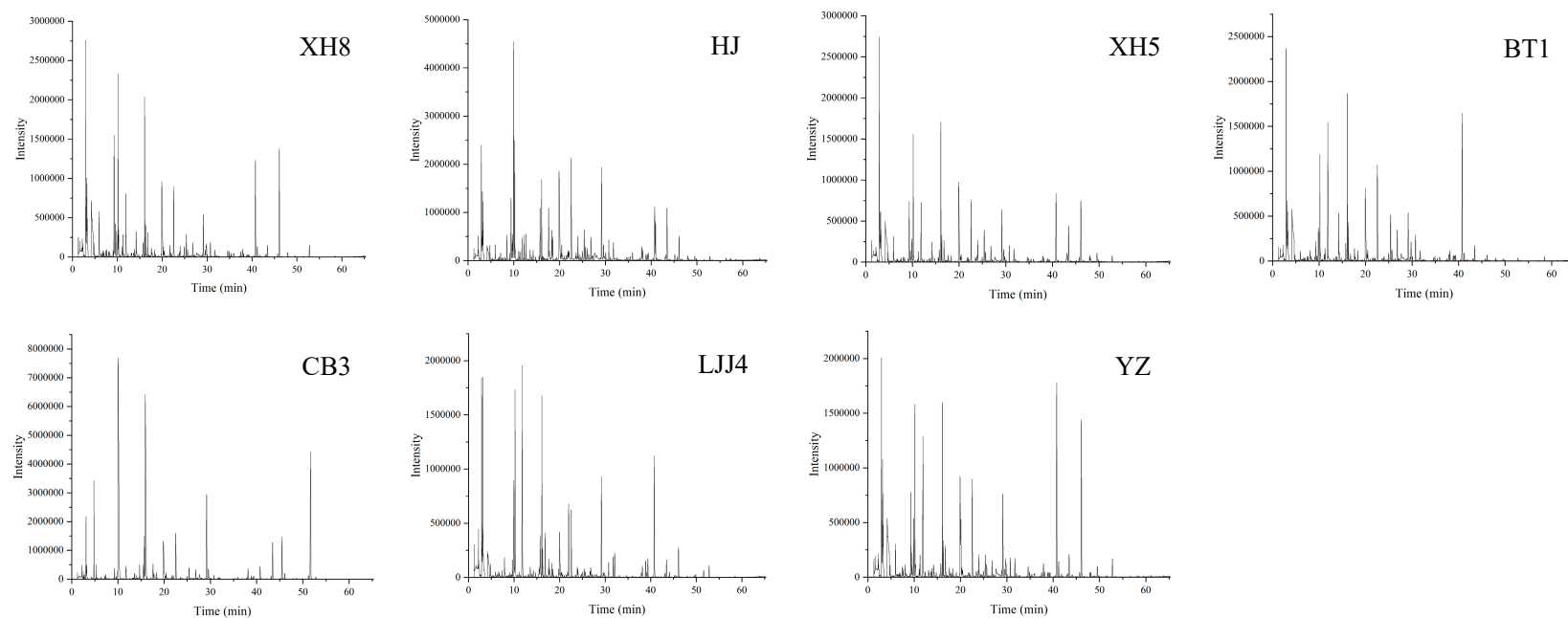

Figure S1.Total ion chromatogram of volatile compounds in seven soy sauce samples

Supplement: Supplementary file 1 [file foods-12-03693-s001.zip › foods-2571463-supplementary.pdf]
